# The Big Nose Pattern at the Second Upper Molar—A Retrospective CBCT Study

**DOI:** 10.3390/dj14050280

**Published:** 2026-05-08

**Authors:** Carol Antonio Dandoczi, Mugurel Constantin Rusu, Răzvan Costin Tudose, Mihail Silviu Tudosie

**Affiliations:** 1 Division of Anatomy, Department 1, Faculty of Dentistry, “Carol Davila” University of Medicine and Pharmacy, 050474 Bucharest, Romania; carol-antonio.dandoczi@drd.umfcd.ro (C.A.D.);; 2 Research Department, “Dr. Carol Davila” Central Military Emergency Hospital, 010825 Bucharest, Romania; 3 Faculty of Medicine, “Carol Davila” University of Medicine and Pharmacy, 050474 Bucharest, Romania

**Keywords:** maxillary sinus, cone-beam computed tomography, pneumatisation, nasal fossa, Big Nose pattern, sinus floor elevation, posterior maxilla

## Abstract

**Background/Objectives**: A marked anteroposterior gradient of nasal fossa pneumatisation over the posterior maxillary alveolar base has been documented at the second premolar level, yet whether this gradient extends to the second upper molar (M2)—the primary site for posterior implant rehabilitation—remains uncharacterised. We aimed to quantify this gradient by classifying pneumatisation patterns above the maxillary alveolar base at M2 (Type 1: pure antral; Type 2: antral with palatine recess; Type 3: Big Nose pattern with combined antral and nasal involvement), assess bilateral symmetry and sex distribution, and compare findings with published second premolar data. **Methods**: A retrospective study was conducted on 165 cone-beam computed tomography scans (330 sides) from a Romanian population. Patterns were classified as Type 1 (pure antral), Type 2 (antral with palatine recess), or Type 3 (Big Nose pattern). Bilateral symmetry was assessed using Cohen’s kappa, and sex differences using Fisher’s exact test. **Results**: Type 1 was observed in 93.3% of sides, Type 2 in 4.2%, and Type 3 in 2.4%. Bilateral symmetry was 98.8% (kappa = 0.904), with all Type 3 cases occurring bilaterally. No significant sex difference was found (*p* = 0.363), although Type 3 showed a non-significant male predominance (OR = 4.55; *p* = 0.305). The Big Nose pattern was 6.8-fold less prevalent at M2 than at the second premolar level. **Conclusions**: A 6.8-fold reduction in Big Nose prevalence from the second premolar (16.2%) to M2 (2.4%) confirms a pronounced anteroposterior gradient in nasal fossa involvement over the posterior maxillary alveolar base—the central finding of this study. At M2, the maxillary sinus dominates exclusively in 97.6% of sides, rendering standard sinus floor elevation highly predictable. The invariable bilaterality of the Big Nose pattern at M2 supports contralaterally symmetrical surgical planning. These findings provide a gradient-based clinical framework: nasal-floor-aware augmentation planning is essential anteriorly (premolar region), whereas standard sinus augmentation protocols are reliably applicable at M2.

## 1. Introduction

Pneumatisation of the midface creates air-filled cavities within the maxilla that may extend inferiorly toward the alveolar process, reducing residual bone height and increasing the risk of perforation during dentoalveolar surgery. The principal cavity is the maxillary sinus (MS), a pyramidal, mucosa-lined space whose medial wall borders the lateral nasal wall and whose ostium communicates with the nasal cavity. Clinically, the MS floor (MSF) is not a simple planar boundary but a variable bony interface shaped by development, function, and remodelling [1]. It may form alveolar recesses that approach or overlap the roots of posterior teeth, creating site-specific patterns of pneumatisation above the alveolar bone. The palatine recess represents a distinct pattern in which sinus pneumatisation extends medially toward the palatal aspect of the alveolar process; this configuration modifies the medial boundary of the MS and should be systematically assessed in presurgical imaging [2].

Within this interface, tooth-related pneumatisation patterns of the MSF are highly heterogeneous. Cone-beam CT studies commonly categorise the relationship between posterior root apices and the MSF as (i) apex inferior to the floor, (ii) apex contacting the floor, or (iii) apex projecting into the sinus, with the latter occurring with notable frequency in molar sites [3]. The maxillary second molar (M2) is particularly relevant: its buccal roots, especially the mesiobuccal root, often demonstrate the smallest vertical distance to the MSF, increasing susceptibility to oroantral communication, Schneiderian membrane violation, and odontogenic sinus complications during extraction, endodontics, and implant osteotomy preparation [3].

Post-extraction remodelling can further accentuate these patterns by combining crestal bone loss with inferior sinus expansion; notably, second molar extraction has been associated with greater pneumatisation than other posterior tooth sites [4,5]. Significantly, however, pneumatised space superior to the posterior alveolar bone is not restricted to the MS: inferior nasal meatus pneumatisation may extend posteriorly and laterally so that the inferior meatus overlies the maxillary alveolar base, mimicking an MSF on two-dimensional imaging and redirecting augmentation planning toward nasal floor elevation rather than classic sinus grafting [2,6]. Given that the MSF augmentation remains a cornerstone intervention for posterior maxillary implant rehabilitation [1,7], a precise description of these pneumatic patterns, particularly around M2, has direct surgical value for oral and maxillofacial surgeons.

To our knowledge, this is the first study to classify pneumatisation patterns above the maxillary alveolar base specifically at the M2 level using this classification system, and the first to provide quantitative data on the anteroposterior gradient of the Big Nose pattern across the posterior maxilla. This gradient—from premolar to molar—has direct implications for site-specific surgical planning and has not previously been characterised. This study aimed to classify and quantify pneumatisation patterns above the maxillary alveolar base at M2 using CBCT, to assess bilateral symmetry and sex distribution, and to compare findings with premolar-level data to define the anteroposterior gradient of sinus and nasal fossa pneumatisation in the posterior maxilla.

## 2. Materials and Methods

A retrospective observational study was conducted on an archived batch of cone-beam computed tomography (CBCT) scans from a Romanian population—the study protocol adhered to the principles of the Declaration of Helsinki [8]. Inclusion criteria were high-quality CBCT scans without distortion or artefact, a complete vertical scan path of the maxilla at the second upper molar (M2) level, and the presence of adjacent or opposing teeth to allow identification of the M2 site in edentulous cases. Patients with the second upper molar present or with clearly identifiable edentulous M2 sites were included. Exclusion criteria included incomplete or unclear scans, distorted anatomical structures, and the impossibility of locating the second upper molar area, as well as scans showing significant sinus pathology (tumours, extensive opacification, or prior surgical intervention in the maxillary sinus region), and cases with impacted or partially erupted second molars that could distort the anatomical relationships under assessment.

All CBCT scans were acquired using an iCAT cone-beam computed tomography system (Imaging Sciences International, Hatfield, PA, USA) operating at 120 kVp and 5 mA, with a field of view of 16 cm × 13 cm and a voxel size of 300 μm. Image analysis and measurements were performed using Planmeca Romexis software (v.3.2.7, Planmeca Oy, Helsinki, Finland). Orthoradial sections at the upper second molar level were used for classification and measurements. All scans were independently classified by two examiners (C.A.D. and M.C.R.). Because the classification involved qualitative categorical variables (Types 1, 2, and 3) rather than continuous measurements, Cohen’s kappa coefficient was used to assess inter-rater agreement. Agreement was excellent (κ = 0.94), and discrepancies were resolved by consensus.

**Figure 1:**
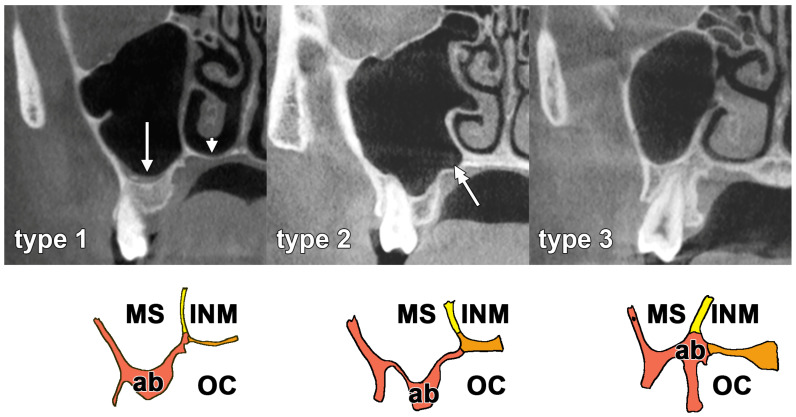
Coronal CBCT slices through the second upper molars depicting the types of pneumatisations above the maxillary alveolar bone. The antral floor (arrow), nasal floor (arrowhead), and palatine recess of the MS (double-headed arrow). Type 1: antral; Type 2: antral, with palatine recess; Type 3: antral and nasal (Big Nose pattern). MS: maxillary sinus; INM: inferior nasal meatus; OC: oral cavity; ab: alveolar bone.

Based on the anatomical relationship between the maxillary alveolar base (MAB) and the overlying pneumatic cavities, three topographic types were identified (Figure 1). Type 1 represented exclusively antral pneumatisation, where the MAB was related superiorly only to the MS; on coronal sections, the lateral nasal wall was aligned with the lateral margin of the palatine process of the maxilla, confirming that the nasal fossa did not extend laterally over the alveolar bone. Type 2 represented antral pneumatisation with a palatine recess; the lateral nasal wall remained aligned with the lateral margin of the palatine process. Type 3, designated as the Big Nose pattern, represented combined antral and nasal pneumatisation, where both the MS and the inferior nasal meatus reached the superior aspect of the MAB (the lateral nasal wall extended superiorly to the alveolar process, indicating that the inferior meatus had pneumatised laterally beyond the palatine process to overlie the alveolar bone). For comparative analyses, Types 1 and 2 were grouped as “pure antral” patterns because the nasal fossa does not directly reach above the alveolar bone.

Statistical analyses were performed using EViews 14 (IHS Markit, London, UK). Descriptive statistics were calculated for each side (n = 330) to characterise the prevalence and distribution of each pneumatisation type. Because the left and right sides are clustered within individuals, inferential analyses comparing pneumatisation patterns between genders were conducted at the subject level (n = 165). For subject-level analyses, each participant was assigned a single pneumatisation type based on the highest type observed on both sides (Type 3 > Type 2 > Type 1); this approach avoids pseudoreplication and reflects clinical planning for the more constrained anatomy. Associations were assessed using chi-square tests or Fisher’s exact tests when expected cell counts were <5. Odds ratios (ORs) with 95% confidence intervals (CIs) were calculated for the Big Nose pattern (Type 3). Statistical significance was set at α = 0.05 for all tests.

## 3. Results

A total of 165 CBCT scans meeting the inclusion criteria were analysed, comprising 67 males (40.6%) and 98 females (59.4%).

### 3.1. Overall Distribution of Pneumatisation Types

A total of 330 sides (165 right and 165 left) were analysed. Type 1 (pure antral) was by far the most prevalent pattern, observed in 308 of 330 sides (93.3%; 95% CI: 90.1–95.6%). Type 2 (antral with palatine recess) was found in 14 sides (4.2%; 95% CI: 2.5–7.0%), while the Big Nose pattern (Type 3) was identified in only eight sides (2.4%; 95% CI: 1.2–4.7%). When Types 1 and 2 were combined as pure antral patterns, 322 of 330 sides (97.6%; 95% CI: 95.3–98.8%) showed pneumatisation exclusively by the MS, with the nasal fossa not reaching the alveolar bone.

### 3.2. Distribution by Side

The distribution of pneumatisation types was notably similar between the right and left sides (Table 1). On the right, Type 1 was present in 155 cases (93.9%), Type 2 in 6 cases (3.6%), and Type 3 in 4 cases (2.4%). On the left, Type 1 appeared in 153 cases (92.7%), Type 2 in 8 cases (4.8%), and Type 3 in 4 cases (2.4%). The McNemar–Bowker test indicated no statistically significant difference in the marginal distributions between the sides (χ^2^ = 2.00, df = 3, *p* = 0.572).

### 3.3. Bilateral Symmetry

Bilateral symmetry was remarkably high, with 163 of 165 individuals (98.8%; 95% CI: 95.7–99.7%) presenting the same pneumatisation type on both sides (Table 2). Cohen’s kappa coefficient was 0.904, indicating almost perfect agreement between the right and left sides. Detailed analysis of bilateral patterns revealed that 153 individuals (92.7%) had bilateral Type 1, 6 individuals (3.6%) had bilateral Type 2, and 4 individuals (2.4%) had bilateral Type 3. Only two individuals (1.2%) exhibited asymmetric patterns; both were female, with Type 1 on the right side and Type 2 on the left. Notably, all four individuals with the Big Nose pattern had bilateral Type 3, suggesting that when nasal fossa pneumatisation extends to the M2 level, it tends to occur symmetrically.

Analysis by gender at the subject level (n = 165; 67 males and 98 females; Table 3) revealed no statistically significant association between gender and the distribution of pneumatisation types (χ^2^ = 2.03, df = 2, *p* = 0.363). Type 1 remained predominant in both males (61/67, 91.0%) and females (92/98, 93.9%). Type 2 was present in 3/67 males (4.5%) and 5/98 females (5.1%). The Big Nose pattern (Type 3) was rare overall (4/165, 2.4%) and occurred in 3/67 males (4.5%) and 1/98 females (1.0%) (OR = 4.55, 95% CI: 0.46–44.68; Fisher’s exact *p* = 0.305). In absolute terms, three of the four subjects with the Big Nose pattern were male.

Bilateral symmetry rates were similar between genders. All 67 male subjects exhibited complete bilateral agreement. Among females, 96 of 98 exhibited bilateral agreement, with two subjects presenting Type 1 on the right and Type 2 on the left; for subject-level analyses, these two cases were classified as Type 2 (maximum type across sides).

## 4. Discussion

### 4.1. Anteroposterior Gradient of Pneumatisation Patterns

The central finding of this study is a pronounced anteroposterior gradient in nasal fossa pneumatisation over the maxillary alveolar base: the Big Nose pattern (Type 3) was 6.8-fold less prevalent at M2 than at the second premolar level, and nasal involvement of any kind was 8-fold less frequent. Direct comparison with Mureșan et al. (2024) at the second upper premolar level, which employed the same classification system in a comparable Romanian population (Table 4), demonstrates that the Big Nose pattern (Type 3) was observed in only 2.4% of sides at M2, compared to 16.2% at PM2, representing a 6.8-fold difference [2]. When considering all patterns involving nasal fossa pneumatisation (Types 3 and 4 at PM2), the disparity is even more pronounced: 19.3% at PM2 versus 2.4% at M2, an 8-fold difference [2]. Conversely, pure antral patterns (Types 1 and 2 combined) were significantly more prevalent at M2 (97.6%) than at PM2 (80.7%) [2].

The prevalence of palatine recesses (Type 2) also differed between regions, being 4.2% at M2 compared to 13.5% at PM2, a 3.2-fold difference, suggesting that medial extension of the MS is more common anteriorly, where the sinus competes with the nasal fossa for space above the alveolar bone [2].

The palatine (palatal) recess of the MS is a key variant of inferior and medial sinus pneumatisation with critical surgical implications. It was defined as a medial–palatal extension of the MSF, creating an additional recess between the antral–palatal wall and the palatal process of the maxilla [2]. Previous authors have often referred to these as palatonasal recesses, emphasising their proximity to the nasal cavity [9,10]. They are reported variably and sometimes over- or miscounted [2]. Importantly, palatal recesses are not necessarily bilateral, emphasising the need for side-specific imaging [2]. Palatal or palatonasal recesses may mimic pathology on imaging and therefore require careful CBCT assessment before interpreting radiolucencies or planning surgery [2]. Their presence influences the choice of approach for sinus augmentation: a palatal sinus lift through the antral–palatal wall can exploit a deep palatal recess and may provide less oedema, less marginal bone loss, and higher postoperative bone density around implants when the buccal wall is thick or scarred [11].

This anteroposterior gradient can be explained by developmental anatomy. The MS begins development in the premolar region during fetal life and expands posteriorly throughout childhood and adolescence. At its anterior limit near the premolars, the sinus competes directly with the lateral expansion of the inferior nasal meatus for pneumatic space above the alveolar bone. As the sinus expands posteriorly, it establishes clear dominance, explaining why the Big Nose pattern is rare at M2. The absence of Type 4 (exclusively nasal) at M2 in our sample, compared to 3.1% at PM2, further supports this concept of progressive posterior sinus dominance [2].

Complementary evidence from Dandoczi et al. (2025), who classified canine fossa (CF) topography at PM1 and PM2, demonstrates the same gradient [12]. At PM1, nasal involvement occurred in 61.8% of sides, whereas at PM2, the CF predominantly bordered the sinus (78.7% Type 1), with residual combined antral–nasal relationships (21.3%) and no isolated nasal type [12]. When considered alongside PM2 alveolar-base topography (nasal involvement 19.3%) and the present M2 findings (2.4%), the combined evidence supports progressive posterior dominance of the MS with decreasing nasal extension into the surgical corridor (Table 5).

Notably, Dandoczi et al. reported that the combined antral–nasal CF configuration at PM2 was associated with leptoprosopic facial morphology (OR 4.71), suggesting that craniofacial form may contribute to the probability of nasal encroachment at site-specific surgical windows [12]. This provides a framework for future work examining whether the M2 Big Nose pattern similarly tracks with craniofacial morphometrics.

Longitudinal CT data confirm that sinus growth is not uniform: the most extensive expansion occurs during the first 8 years of life, and maximal values for all diameters and volume are reached by the end of the 16th year, with no bilateral dimorphism but clear gender-related differences after age 8 [13]. In a comprehensive review, it was emphasised that the paranasal sinuses constitute an integral part of a common mucosal organ, and that postnatal MS expansion is intimately linked to the alveolar process morphology and eruption sequence of the posterior teeth [14]. Additionally, Lee et al. (2020) demonstrated through 3D reconstruction that the MS gradually transforms from an ellipsoidal structure at its rudimentary phase into a pyramidal shape when fully matured, with the most rapid volumetric expansion occurring in the first 4–5 years, providing a morphological basis for the progressive posterior dominance observed in the present study [15].

The anteroposterior gradient described here has important implications for understanding post-extraction remodelling. Cavalcanti et al. (2018) reported that tooth loss in the posterior maxilla favourably promotes sinus pneumatisation [16]. That localisation of sinus pneumatisation at molar roots appears to predict a greater need for sinus lift surgery, with 54% of such cases presenting residual bone heights below 5 mm [16]. Furthermore, other authors have demonstrated that the degree of MS pneumatisation within the alveolar process is significantly greater in younger adults (18–34 years) than in older groups, decreasing gradually with age, with no sex- or side-related differences [17]. This age-dependent pattern may interact with the anteroposterior gradient, such that younger patients with premolar-level Big Nose patterns could theoretically exhibit different remodelling trajectories than older cohorts following tooth extraction.

This finding aligns with a recent CBCT study that examined changes in MS volume across different craniofacial skeletal patterns and found that MS volume was significantly larger in males than in females, although the sagittal skeletal pattern (Class I, II, or III) did not significantly affect overall MS volume [18]. Similarly, Lee and Park (2022) demonstrated that the MS tends to be wider in mesoprosopic face types and taller in hyperleptoprosopic types, emphasising that facial index may influence the distribution of pneumatisation at specific sites [19].

### 4.2. Correlation with Other Classification Systems

Haj Yahya et al. (2021) evaluated the Big Nose variant on CBCT by locating the mesiodistal nasal–sinus border (“classes”) and then describing the tooth-by-tooth vertical relationship (“divisions”) from canine to first molar [20]. In 321 CBCT scans, they reported Class 3 (nasal–sinus border distal to the mesial edge of the first molar) in 17.9% of hemi-maxillae [20]. The apparent discrepancy with our 2.4% prevalence at M2 is explained by non-equivalent endpoints: Haj Yahya et al. operationalised the Big Nose variant as a posterior shift in the nasal–sinus border extending only through the first molar region, whereas the present study records a specific M2-level floor-source configuration. Therefore, their Class 3 likely includes a spectrum of posterior nasal extension patterns, only a subset of which would manifest as strict M2-level nasal-floor dominance (Type 3) in our scheme [20]. Notably, their “divisions” findings still support posterior sinus predominance: the MS alone was superior to the first molar in 85.6% of cases (Division C), consistent with our observation that the M2 level is overwhelmingly pure antral [20].

A recent narrative review on MS hypoplasia further contextualises these classification discrepancies [21]. It was highlighted that inferior meatus pneumatisation represents a lateral expansion of the nasal cavity requiring nasal floor elevation when placing implants in the posterior maxilla. Accurate diagnosis of such variants requires 3D radiographic techniques such as CBCT [21]. This supports the view that two-dimensional approaches may under- or overestimate the true prevalence of the Big Nose pattern depending on the classification system employed. In addition, Shetty et al. (2021) developed and validated a novel CBCT-based index to evaluate surgical sites before sinus lift procedures, incorporating six key parameters: lateral wall thickness, sinus membrane thickness, septations, root-membrane relationships, alveolar antral artery visibility, and sinus pathologies [22]. Their index demonstrated good interrater agreement (Fleiss kappa = 0.85) and a highly significant correlation with surgical difficulty scores, reinforcing the importance of systematic CBCT-based presurgical assessment in sinus augmentation planning [22].

### 4.3. Sinonasal Anatomical Interactions

Several studies have evaluated how sinus pneumatisation and nasal variations shape posterior nasal and maxillary morphology. Holton et al. (2013) showed that MS volume is positively correlated with nasal cavity volume, not inversely, and that sinus volume is especially related to internal nasal breadth [23]. This suggests that in Big Nose configurations, a broad internal nasal cavity may coexist with substantial MS volume, with accommodation occurring through shape changes and lateral displacement rather than a simple volume trade-off.

In a study of the posterior superior MS (PSMS), the authors showed that increased PSMS pneumatisation is linked to a narrower upper posterior nasal cavity [24]. Therefore, while inferior meatus pneumatisation pushes the nasal cavity downwards and laterally, superior/posterior maxillary pneumatisation generally reduces nasal width in the upper posterior area, highlighting a complex three-dimensional interaction between sinus development and nasal cavity expansion.

Multiple CBCT studies have examined the relationships among nasal septal deviation, concha bullosa, Haller cells, and inferior sinus pneumatisation, but generally fail to demonstrate a consistent effect of septal deviation or concha bullosa on overall MS volume or on inferior sinus pneumatisation [25,26,27]. Göksel and Güler (2023), however, found significant associations between Haller cells, nasal septum deviation, and alveolar and palatal process pneumatisation of the MS, suggesting that some sinonasal variants may indirectly influence the extent of pneumatisation inferiorly toward the alveolar base [26].

Data from Guidugli et al. (2020) add a volumetric perspective: in a 3D segmentation study of 100 head CT-scans, the volumes of the frontal, sphenoid, and maxillary sinuses were significantly intercorrelated in both sexes, and the MS was the least asymmetric of the three paranasal sinus types, supporting the bilateral symmetry of sinus morphology observed in the present study [28]. A large-scale CBCT analysis in a Saudi population of 1018 scans further confirmed that sinus dimensions (width, length, area, perimeter) were significantly larger in males, decreased progressively after the fifth decade, and demonstrated strong bilateral correlations (rho > 0.55), reinforcing the concept of constitutional determination of pneumatisation patterns across populations [29].

### 4.4. Clinical Implications: Imaging and Surgical Considerations

The clinical implications of the regional differences described above are significant. At the M2 level, surgeons can approach the MSF with high confidence that the alveolar bone is exclusively antral (97.6% of cases), making standard MSF elevation (MSFE) procedures predictable. In contrast, at the PM2 level, nearly one in five cases (19.3%) involve the nasal fossa and require careful preoperative assessment to avoid inadvertent entry into the nasal cavity during palatal approaches [2]. Large CBCT series have shown that the maxillary posterior region is the most common site of positioning errors (51.6% of all implants with errors), particularly thread exposure and excessive proximity to the sinus [30]. This underscores the need for careful 3D planning and the use of CBCT in the M2 zone [30].

When a posterior nasal cavity extension is present, it can fundamentally invert expected relationships between the sinus and nasal floors and lead to inadvertent nasal penetration during posterior maxillary implant surgery. Park et al. (2020) demonstrated that two-dimensional assessment can misclassify nasal-floor penetration as sinus-floor penetration; on panoramic radiographs, some implants appeared to breach the MSF, yet CBCT clarified that they actually penetrated the nasal floor because the pneumatised inferior meatus extended posteriorly into the molar region [31]. Patients with nasal-floor penetration had a wider nasal cavity and a narrower sinus cavity than those with true sinus-floor penetration [31].

Yeom et al. (2023) provided a complementary perspective, identifying two recurring panoramic findings that may suggest posterior nasal cavity extension: (i) the hard palate line positioned inferior to or at the level of the antral floor line, and (ii) a triangular bony area between the lateral nasal wall and the medial MS wall that may create a false impression of abundant residual bone [32]. Three-dimensional imaging is recommended when panoramic cues raise suspicion [32].

Regarding clinical sequelae, Park et al. (2020) reported that nasal floor penetration does not necessarily result in persistent sinonasal morbidity [31]. With a mean follow-up of approximately 14 years, implant survival remained high, with losses attributed to peri-implantitis rather than nasal complications; nasal endoscopy showed that apical portions of the implants were covered by thin mucosa without inflammatory changes [31]. Yeom et al. (2023) similarly noted that nasal cavity penetration can be asymptomatic for prolonged periods, although limited perforation may remain clinically stable [32].

When a posterior nasal cavity extension is identified preoperatively, the augmentation strategy may need to shift from MSFE to a nasal floor elevation approach. Park et al. (2023) reported successful implant placement via simultaneous nasal floor augmentation in such a case, with careful limitation of the lateral bony window and extent of elevation to minimise the risk of inferior turbinate contact or postoperative changes in nasal airflow [6]. Histologic assessment indicated vital bone formation at the grafted site, supporting the feasibility of this approach for selected patients with severe posterior maxillary atrophy and posterior nasal cavity extension [6].

In a systematic review, the authors pooled all available evidence on implants placed after nasal floor elevation. They reported a weighted survival rate of 97.64% over a mean follow-up of 32.2 months, with no statistically significant differences between one-stage and two-stage approaches or among different grafting materials, supporting nasal floor augmentation as a viable alternative when the Big Nose pattern precludes standard MSFE [33]. For the majority of M2-level cases with purely antral anatomy, the MSFE procedure remains highly predictable. A recent systematic review and meta-analysis by Chen et al. (2024) comparing non-grafted versus grafted MSFE found no significant difference in short-term implant survival rates and a low pooled complication rate, confirming the technique’s maturity and reliability [34]. Furthermore, Hsu et al. (2022) conducted a comprehensive systematic review of SFE complications across 4411 procedures and noted that perforation of the Schneiderian membrane (the most frequent intraoperative complication) occurs in approximately 19–30% of lateral-approach cases; however, when properly managed, it does not significantly compromise implant survival [35]. Diaz-Olivares et al. (2021) confirmed these findings in a systematic review and meta-analysis, reporting that Schneiderian membrane perforation during MSFE procedures with the lateral approach is not a risk factor for dental implant survival (*p* = 0.229), with survival rates of 97.68% under perforated membranes versus 98.88% under intact membranes [36]. This evidence reinforces that even when complications arise at the M2 level, long-term outcomes remain favourable, particularly given the anatomical predictability documented in the present study. Supporting this, Rizzo et al. (2022) in a cross-sectional analysis of 590 dental implants, reported that 74.4% of implants were poorly positioned, with the posterior maxilla being the most frequently affected region, further underscoring the need for careful 3D planning [37].

Clinical framework. Taken together, the present data support the following gradient-based preoperative decision framework for posterior maxillary implant planning. At the M2 site, the pneumatisation pattern is pure antral in 97.6% of sides; standard CBCT assessment of residual bone height and sinus floor morphology is sufficient, and standard sinus floor elevation protocols are appropriate. At the PM2 site, nasal fossa involvement is present in approximately 19% of sides; preoperative CBCT should explicitly evaluate whether the inferior nasal meatus overlies the alveolar base, as identification of the Big Nose pattern redirects augmentation from sinus floor elevation to nasal floor elevation. When the Big Nose pattern is confirmed unilaterally, contralateral involvement should be anticipated given the nearly universal bilateral symmetry observed across both M2 and PM2 datasets. Panoramic cues—such as the hard palate line lying at or below the apparent antral floor, or a triangular bony area between the lateral nasal and medial sinus walls—should prompt three-dimensional CBCT confirmation before surgical intervention.

### 4.5. Bilateral Symmetry and Sex Distribution

Both the present study and Mureșan et al. (2024) demonstrated strong bilateral symmetry [2]. In our M2 cohort, 98.8% of individuals presented the same pneumatisation type on both sides (Cohen’s kappa = 0.904). Importantly, all four individuals with the Big Nose pattern had bilateral Type 3, indicating that when nasal fossa pneumatisation reaches M2, it occurs symmetrically—a finding with direct surgical relevance: Type 3 on one side should prompt contralateral assessment. Neither study found a statistically significant association between sex and pneumatisation type. The observed numerical predominance of males among Type 3 cases at M2 (3 of 4; OR = 4.55; 95% CI: 0.46–44.68; *p* = 0.305) must be interpreted with extreme caution: the extremely wide confidence interval and the very small absolute number of Type 3 subjects (n = 4) preclude any meaningful inference about sex-related biological predisposition. This finding should not be generalised beyond the present sample.

The strong bilateral symmetry observed here aligns with broader volumetric data. Guidugli et al. (2020) reported that the MS was the least asymmetric among all paranasal sinuses, with an inverse correlation between sinus volume and asymmetry index, meaning larger sinuses tend to be more symmetric [28]. From a forensic and morphometric perspective, Ghavate et al. (2024) found that MS volume, length, and width showed statistically significant sex differences, with male sinuses consistently larger [38]. Bilateral measurements within individuals remained strongly correlated [38]. Nevertheless, population-specific variability exists: in a study of a Nepalese population, no significant sexual dimorphism in MS dimensions was observed, with the right and left sinuses showing no paired differences in either sex [39]. These findings collectively suggest that while absolute sinus dimensions may vary across populations and between sexes, the bilateral symmetry of pneumatisation patterns, and therefore the constitutional determination of variants like the Big Nose pattern, appear to be a robust finding.

### 4.6. Hypothesis-Generating Considerations

The term “Big Nose” is used here as a descriptive shorthand for the radiographic topography and does not imply a proven functional phenotype. Because the present study did not quantify nasal cavity dimensions, airflow, or sinonasal physiology, any physiological interpretation must be considered hypothesis-generating. A plausible anatomical model is that, in a small subset of individuals, the posterior nasal cavity volume and/or the inferior meatus calibre are relatively expanded, reducing the space available for MS pneumatisation at the M2 level.

Future work should test this hypothesis by integrating CBCT-based craniofacial morphometrics with dedicated nasal airway assessments (e.g., nasal cavity and turbinate volumes, septal alignment, rhinomanometry, and/or computational fluid dynamics) and evaluating potential confounders such as age, facial type, and ethnogeographic background.

This model receives indirect support from developmental studies. The link between MS dimensions and midface parameters during postnatal growth shows that the MS develops alongside the surrounding skeletal structure, and that sinus volume correlates with upper facial height and midfacial width [40]. Furthermore, Maspero et al. (2020) demonstrated that the sinus mainly develops vertically during the pubertal growth peak [41]. That growth timing varies by gender, with female sinuses beginning to develop earlier but ultimately being smaller than those in males [41]. These findings imply that the final shape of the alveolar base, including the presence or absence of a Big Nose pattern, results from a complex interaction between nasal cavity expansion, sinus pneumatisation rate, and craniofacial growth trajectory.

### 4.7. Limitations

Several limitations should be acknowledged. The retrospective single-centre design and Romanian population limit generalisability; pneumatisation patterns may vary across ethnic groups due to differences in craniofacial morphology and genetic factors. The low prevalence of Type 3 (4 subjects) substantially limits statistical power for subgroup analyses, and the wide confidence interval around the sex-related odds ratio (0.46–44.68) precludes definitive conclusions regarding male predominance.

The study was purely morphological and did not include functional airway assessment or craniofacial morphometrics; therefore, the hypothesis that the Big Nose pattern represents a respiratory adaptation remains controversial. Additionally, the cross-sectional design cannot capture developmental changes over time, and using an archived clinical CBCT database may introduce selection bias, although strict inclusion criteria partially mitigate this concern.

It should also be noted that the relationship between root apices and the sinus floor is population-dependent. Akotiya et al. (2024) reported in a Central Indian cohort that the mesiobuccal root of the second molar had the shortest distance to the MS floor among all posterior teeth [42], echoing the findings at M2 in our Romanian sample and suggesting that while absolute distances may vary across ethnic groups, the M2 region consistently represents a site of maximal sinus–alveolar proximity. Multi-centre studies across diverse populations will be necessary to determine whether the prevalence gradient of the Big Nose pattern is a universal anatomical principle or one subject to significant ethnogeographic variation.

## 5. Conclusions

The central finding of this study is a pronounced anteroposterior gradient in nasal fossa pneumatisation over the posterior maxillary alveolar base: the Big Nose pattern (Type 3) was 6.8-fold less prevalent at M2 (2.4%) than at the second premolar level (16.2%), and total nasal involvement was 8-fold lower (2.4% vs. 19.3%). This gradient reflects the progressive posterior dominance of maxillary sinus pneumatisation and is the first to be quantified with comparable methodology across the posterior maxilla.

At M2, pure antral pneumatisation is present in 97.6% of sides, supporting the predictability of standard sinus floor elevation at this site. The Big Nose pattern is rare but invariably bilateral, indicating that its identification on one side reliably predicts contralateral involvement. These data support a gradient-based surgical planning approach: standard sinus augmentation protocols are highly applicable at M2, while CBCT-guided, nasal-floor-aware assessment is essential at the premolar level. Sex distribution showed no significant association with pneumatisation type; the numerically observed male predominance among Type 3 cases must not be over-interpreted given the very small subgroup size.

## Figures and Tables

**Table 1:** Distribution of pneumatisation types by side.

Type	Right Side	Left Side	Combined
Type 1 (pure antral)	155 (93.9%)	153 (92.7%)	308 (93.3%)
Type 2 (palatine recess)	6 (3.6%)	8 (4.8%)	14 (4.2%)
Type 3 (Big Nose)	4 (2.4%)	4 (2.4%)	8 (2.4%)
Pure antral (Types 1 + 2)	161 (97.6%)	161 (97.6%)	322 (97.6%)

**Table 2:** Contingency table showing bilateral distribution of pneumatisation types (n = 165).

	Left Type 1	Left Type 2	Left Type 3
Right Type 1	153	2	0
Right Type 2	0	6	0
Right Type 3	0	0	4

**Table 3:** Distribution of pneumatisation types by gender (subject-level, n = 165). *p*-values from Fisher’s exact test.

Type	Male (n = 67)	Female (n = 98)	*p*-Value
Type 1 (pure antral)	61 (91.0%)	92 (93.9%)	0.550
Type 2 (palatine recess)	3 (4.5%)	5 (5.1%)	1.000
Type 3 (Big Nose)	3 (4.5%)	1 (1.0%)	0.305

**Table 4:** Comparison of pneumatisation patterns at M2 (present study) and PM2 [2].

Type	M2 (n = 330)	PM2 (n = 290)	Ratio
Type 1 (pure antral)	308 (93.3%)	195 (67.2%)	—
Type 2 (palatine recess)	14 (4.2%)	39 (13.5%)	×3.2 at PM2
Type 3 (Big Nose)	8 (2.4%)	47 (16.2%)	×6.8 at PM2
Type 4 (exclusively nasal)	—	9 (3.1%)	—
Pure antral (Types 1 + 2)	322 (97.6%)	234 (80.7%)	—
Nasal involvement	8 (2.4%)	56 (19.3%)	×8.0 at PM2

**Table 5:** Harmonised comparison of antral and nasal relationships across the canine fossa (CF), PM2, and M2 (side-based frequencies). Note: Canine fossa types are defined by the CF’s medial relationship to the MS and/or nasal fossa, whereas PM2/M2 types are determined by the relationship of the maxillary alveolar base to overlying pneumatic cavities; alignment is conceptual.

Site/Study (n, Sides)	Antral Only	Combined	Nasal Only
CF, PM1 (n = 178)	68 (38.2%)	88 (49.4%)	22 (12.4%)
CF, PM2 (n = 178)	140 (78.7%)	38 (21.3%)	0 (0.0%)
Alveolar base, PM2 (n = 290)	234 (80.7%)	47 (16.2%)	9 (3.1%)
Alveolar base, M2 (n = 330)	322 (97.6%)	8 (2.4%)	0 (0.0%)

## Data Availability

The datasets used and analysed during the current study are available from the corresponding author upon reasonable request.

## Abbreviations

The following abbreviations are used in this manuscript:

CBCT	cone-beam computed tomography
CF	canine fossa
CI	confidence interval
CT	computed tomography
df	degrees of freedom
M2	maxillary second molar (upper second molar)
MAB	maxillary alveolar base
MS	maxillary sinus
MSF	maxillary sinus floor
MSFE	maxillary sinus floor elevation
OR	odds ratio
PM1	maxillary first premolar
PM2	maxillary second premolar
PSMS	posterior superior maxillary sinus
